# Comparative Analysis of Antibacterial Efficacy and Flow Characteristics of Three Root Canal Sealers

**DOI:** 10.7759/cureus.68659

**Published:** 2024-09-04

**Authors:** Sazgar S Qasim Al-Hawezi, Bnar A Perdawd, Lanja A Ali, Niaz H Hamasaeed, Mohammed M Al Moaleem

**Affiliations:** 1 Conservative Dentistry and Endodontics, Hawler Medical University, College of Dentistry, Erbil, IRQ; 2 Conservative Dentistry, Hawler Medical University, College of Dentistry, Erbil, IRQ; 3 Prosthetic Dental Science, Jazan University, College of Dentistry, Jazan, SAU

**Keywords:** root canal sealer, antimicrobial, flow test, guttaflow2, ah plus, endofill

## Abstract

Background: Oral microflora containing microorganisms is responsible for the majority of orodental diseases as well as post-endodontic treatment failure. Even in the ideal root canal treatment cases, a small number of viable bacteria will remain in the dentinal tubule hence come the role of root canal sealers, which must offer and possess some degree of antimicrobial activity alongside adequate flowability to ensure excellent sealing of all lateral dentinal tubule canals to prevent the possibility of future reinfection. The present study aimed to examine the antibacterial efficacy against *Enterococcus faecalis* (*E. faecalis*) using an agar diffusion test (ADT) at different time intervals, as well as the flow characteristics of three different root canal sealers: Endofill (Produits Dentaires SA,* *Vevey, Switzerland), AH Plus (Dentsply De Trey GmbH, Konstanz, Germany), and the newly introduced GuttaFlow2 (Coltène/Whaledent, Altstätten, Switzerland).

Materials and methods: The antibacterial activity and flow characteristics of three root canal sealers, Endofill, AH Plus, and GuttaFlow2, were tested using ADT at three different time intervals (24 hours, 48 hours, and seven days). For this purpose, *E. faecalis* strains were used as the pathogen in this study. Flow characteristics were done using the standard flow test protocol recommended for endodontic sealers. Data were analyzed using one-way ANOVA and the Tukey post hoc test, with a p-value < 0.05 considered statistically significant.

Results: The results for antibacterial activity showed a statistically significant difference between Endofill and the other groups (p < 0.05). The antibacterial activity of Endofill increased over time from 24 hours to 48 hours and seven days. AH Plus demonstrated antibacterial activity only within the first 24 hours of mixing, while GuttaFlow2 showed no inhibition zones against *E. faecalis*. Regarding the flow test results, the Endofill group recorded the lowest flow values compared to GuttaFlow2 and AH Plus, which was statistically significant (p < 0.05). There was no statistically significant difference between GuttaFlow2 and AH Plus for flow values.

Conclusion: Endofill demonstrated the highest antibacterial activity at all time intervals, while GuttaFlow2 showed no antimicrobial activity. AH Plus exhibited antimicrobial effects only within the first 24 hours of mixing. In terms of flow values, Endofill had the lowest flow, whereas GuttaFlow2 and AH Plus had the highest flow values.

## Introduction

Sealers are a type of dental material used as a thin, tacky paste that functions as a lubricant and luting agent during root canal obturation [1]. They allow the core obturation material, such as gutta-percha points or other rigid materials, to easily slide in and become fixed in the prepared canal [2]. Additionally, sealers must achieve two main goals: creating an apical seal and filling the root canal without incorporating voids within the filling material [1,2]. The primary function of endodontic sealing materials is to obstruct any possible route for pathogens and to prevent bacterial leakage. Furthermore, root canal sealers should prevent microbial reinfection by sealing both the lateral and principal root canals, as well as by preventing any potential microbial colonization. Additionally, endodontic sealers should effectively fill the space between the gutta-percha and the dentinal walls of the root canal [1].

Today, there are seven categories of sealers used in dental clinics, which can be classified based on their basic chemical composition: zinc oxide eugenol (ZOE)-based sealer, silicone-based sealer, mineral trioxide aggregate-based sealer, epoxy-based sealer, bioceramic-based sealer, calcium hydroxide-based sealer, and glass ionomer-based sealer [2]. Bacteriostatic activity is considered one of the most important criteria for root canal sealers; at a minimum, they should not promote bacterial growth [3]. The most commonly used sealer is the ZOE sealer, which has been utilized for decades due to its adequate physicochemical properties [3]. However, leakage and contamination of the root canal are often attributed to the eugenol or zinc oxide leaching through ongoing hydrolysis, which can lead to future complications [4].

Another type of sealer is AH Plus (Dentsply De Trey GmbH, Konstanz, Germany), an epoxy resin-based endodontic sealer. It has advantages such as ease of handling, superior wettability of the root canal dentin and gutta-percha interface, and excellent sealing properties. Epoxy resin-based sealers can easily penetrate dentinal tubules and create monoblocks between the canal filling material and root canal dentin, making them one of the most important types of endodontic sealers [5].

In addition to the aforementioned sealers, GuttaFlow2 (Coltène/Whaledent, Altstätten, Switzerland), a silicone-based sealer, has been highlighted for its superior biocompatibility, exceptional sealing ability, and adaptability to the root canal dentin walls. These properties are attributed to its high flowability and the fact that it expands by 0.2% when it sets [6]. GuttaFlow2 is composed of gutta-percha powder, polydimethylsiloxane, silver particles, and bioactive substances that stimulate the release of natural repair by-products, assisting in the regeneration of periapical tissues [6].

Among the many microorganisms, *Enterococcus faecalis* is one of the most frequently observed in the root canals of teeth with unsuccessful root canal treatments. Its pathogenicity ranges from mild conditions to potentially fatal disorders in medically compromised individuals, such as infections in root canal-treated teeth with persistent apical periodontic lesions. Furthermore, like many other pathogenic microorganisms, *E. faecalis* adheres efficiently to biotic and abiotic surfaces, secreting a protective extracellular matrix that leads to the formation of a multilayer antibiotic-resistant biofilm [7].

To assess the antibacterial activity of endodontic sealers, various testing procedures such as the direct contact test, agar diffusion test (ADT), and time-kill assay have been used [8]. The ADT technique has been extensively employed to examine the antimicrobial efficacy of dental medications and materials, allowing direct comparisons of endodontic sealers against the tested microorganisms [8].

Thus, the current study was designed to observe the antibacterial efficacy of three root canal sealers against *E. faecalis* using the ADT: Endofill (Produits Dentaires SA, Vevey, Switzerland) (a ZOE-based sealer), AH Plus, and GuttaFlow2. The flow characteristics of these sealers were also studied at three different time intervals. The null hypothesis is that there are no significant differences between the tested sealers across different time periods.

## Materials and methods

Study design and grouping

**Figure 1:**
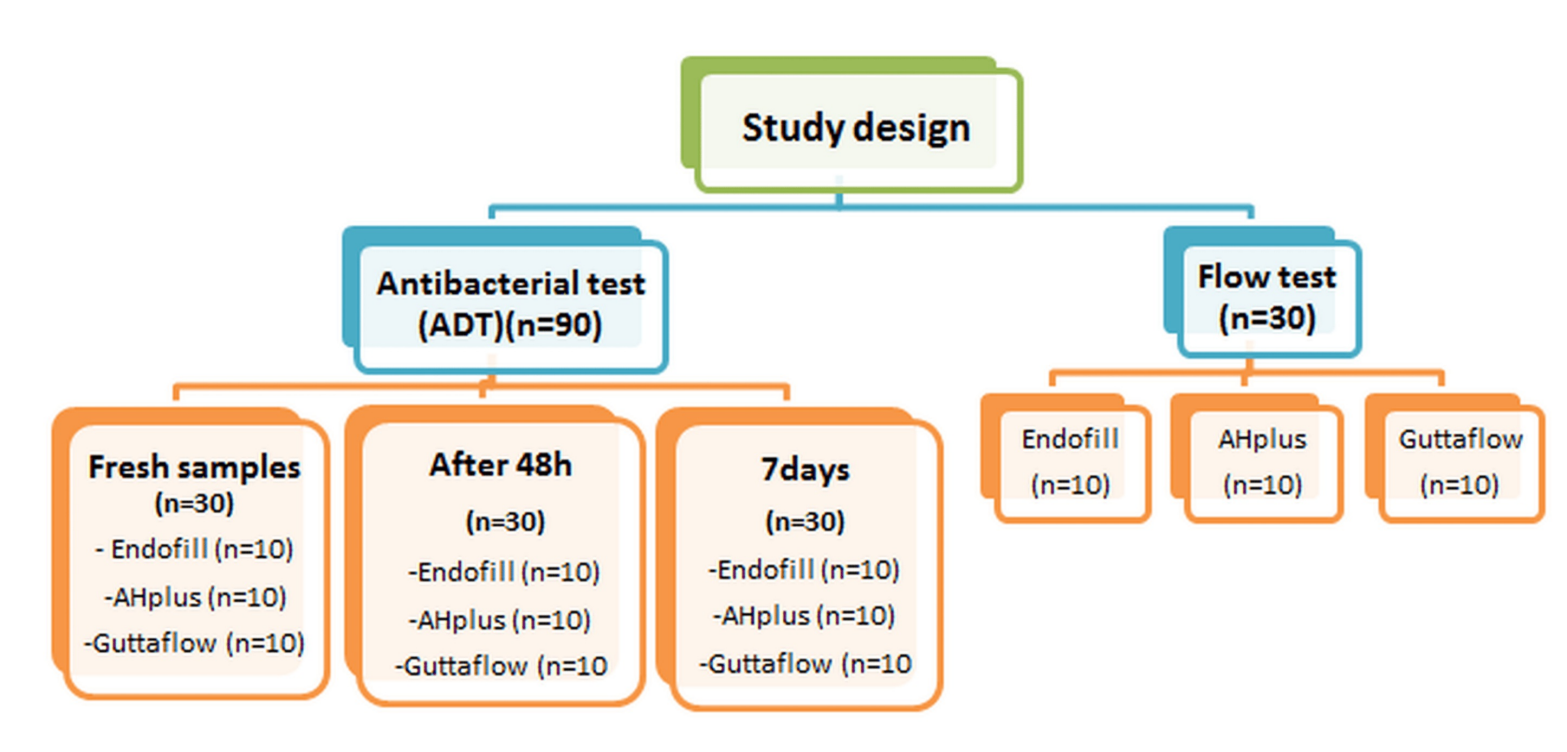
Study design. ADT: agar diffusion test.

A total of 120 samples (90 for the antimicrobial test and 30 for the flow test) were used in this laboratory experimental in vitro study, as shown in Figure 1. The samples were divided into three groups based on the endodontic sealers used and the time intervals for measurement or evaluation. Three endodontic root canal sealers were tested in the present study: Endofill, a ZOE-based sealer; AH Plus, a resin-based sealer; and GuttaFlow2, a silicone-based root canal sealer. The sealer samples were prepared according to the manufacturer’s instructions.

G*Power (version 3.1.9.7, 2020, Heinrich Heine University Düsseldorf, Düsseldorf, Germany) was used to approximate the specimen sizes while assuming a three-group assessment. The alpha was set at 0.05 with a power of 85%, and an effect size of 0.40 was used to calculate the sample size. A total sample size of 90 samples was initiated to be enough.

Sample testing

Antibacterial Test

The pathogen ATCC 29212 *E. faecalis* from a reputable central medical culture center was reactivated by transfer into brain heart infusion (BHI) broth, followed by incubation at 37ºC for 24 hours. For the inoculum, the culture in BHI broth incubated at 37ºC for 18 hours was used to standardize the final concentration to 1.5 × 10^8 cells/mL, equivalent to the 0.5 standard of the McFarland scale, using a spectrophotometer at a wavelength of 630 nm [9].

The testing procedure was conducted using agar diffusion on a double-layered plate. The base layer of the plate consisted of 40 mL of sterilized Sabouraud agar (SA). On each agar plate, four wells, each with a radius of 3 mm and a depth of 5 mm, were created at equal distances. The freshly mixed endodontic sealers were placed into each well. Next, 0.5 mL of the bacterial suspension, standardized to the McFarland scale, was incorporated into 15 mL of SA as the secondary layer. After incubating the agar plate at 37ºC for 24 hours, the diameter of all inhibition zones around each well was measured using a millimeter ruler with an accuracy of 0.5 mm. The mean diameter of the inhibition zones was analyzed statistically to assess the antimicrobial activity of the tested sealers [9]. This procedure was repeated after 48 hours and seven days of mixing the experimental sealers.

Flow Test

**Figure 2:**
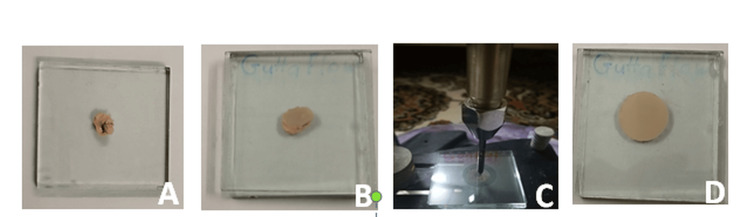
Flow test. (A) Tested sealer placed over glass coverslip. (B) The second glass coverslip placed over the tested sealer and the first glass coverslip. (C) These coverslips were then placed under a constant weight. (D) The tested sealer sample formed an expanding disc.

A predetermined volume of 0.05 mL of the mixed root canal sealer was dropped onto a 2 mm thick flat-surfaced glass plate. Three minutes after the onset of mixing, a second flat-surfaced glass plate of the same thickness was placed on the sealer, followed by the application of a 100 g weight, making a total mass of 120 g. Ten minutes from the start of mixing, the 120 g weight was removed [10], as shown in Figure 2.

For the data collection process, a digital caliper (Mitutoyo, Kawasaki, Japan) with a precision of 0.01 mm was used to measure the minimum and maximum diameters of the sealer disc. Any disc that did not show a uniform circular pattern was excluded, and a new sample was prepared, with the testing procedure repeated. Ten samples were taken for each sealer [10].

Statistical analysis

The data were organized in an Excel sheet (Microsoft Corporation, Redmond, WA) and subjected to statistical analysis. A normality test was conducted to check the data distribution, and the Shapiro-Wilk test showed that the data for all variables were normally distributed.

The data were calculated as mean and standard deviation at different time periods for the ADT and flow tests. The data were analyzed using one-way ANOVA and the Tukey post hoc test with SPSS version 28 (IBM Corp., Armonk, NY). The significance level was set at p < 0.05.

## Results

**Figure 3:**
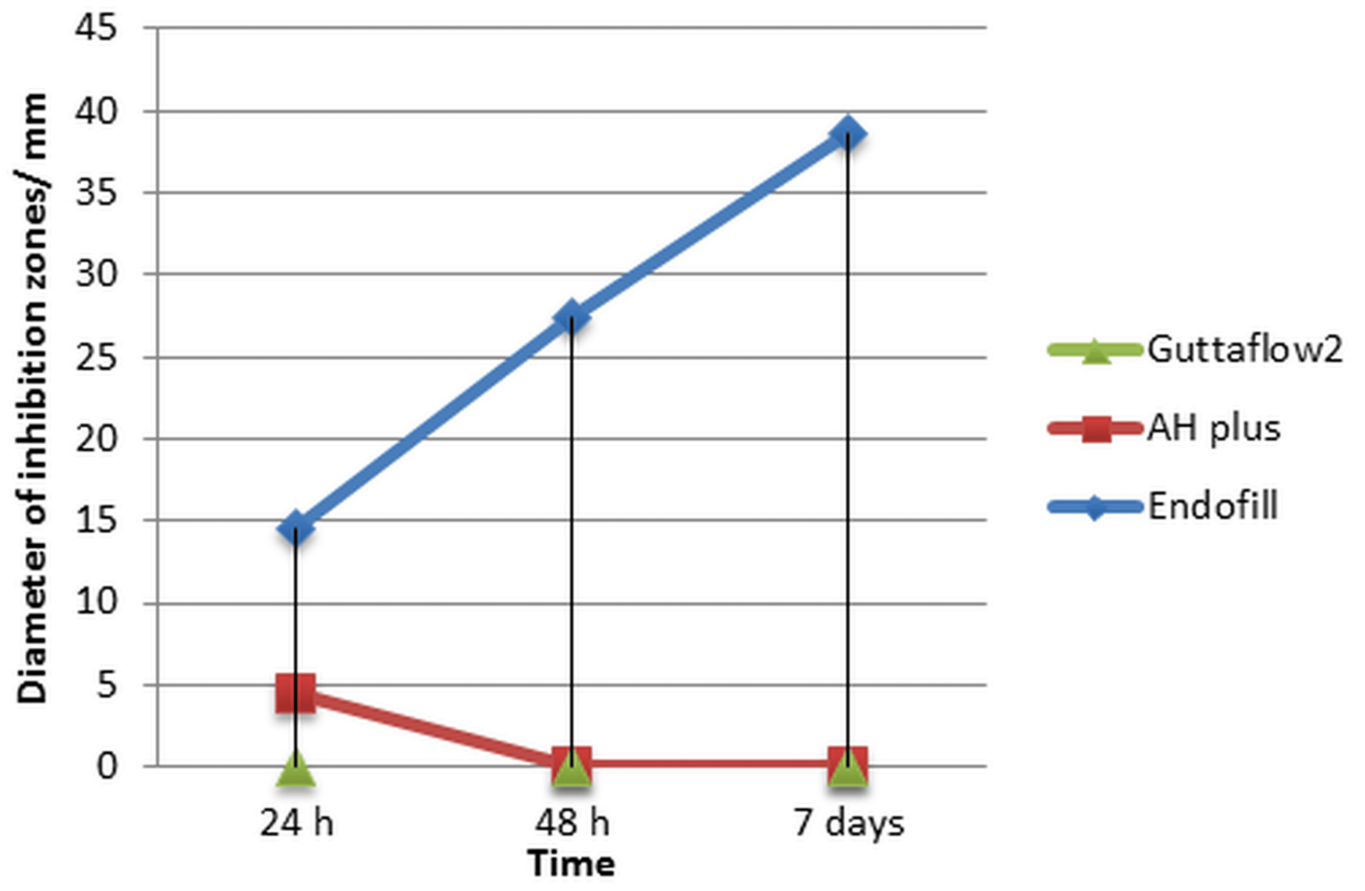
Diameter of inhibition zones of sealer groups at different time intervals.

There was a statistically significant difference among the study groups regarding the antibacterial test at the first 24 hours after sealer mixing (p < 0.000). However, no significant difference was found between the AH Plus and GuttaFlow2 groups after 48 hours and seven days of mixing (set samples) (p = 1.000). The post hoc test showed a statistically significant difference between the set samples of the Endofill sealer group compared to the set samples of both the AH Plus and GuttaFlow2 sealer groups at 48 hours and seven days after mixing (p < 0.05), as shown in Figure 3.

The results of the ADT at the first 24 hours after mixing the sealers showed that the mean values of the tested groups were significantly different, according to ANOVA and post hoc tests. The samples of Endofill sealer recorded the largest diameter of inhibition zones (14.500 mm), followed by the AH Plus sealer (4.500 mm), and Guttaflow2 recorded no inhibition zones, as shown in Table 1 for the ADT.

**Table 1:** ANOVA test of inhibition zone by agar diffusion test.

Sample	N	Mean inhibition zone/mm	SD	S. error	95% confidence interval for mean		Min	Max
					Lower bound	Upper bound		
Endofill, 24 hours	10	14.500	0.5477	0.2236	13.9252	15.0748	14.00	15.00
Endofill, 48 hours	10	27.333	0.8165	0.3333	26.4765	28.1902	26.00	28.00
Endofill, 7 days	10	38.666	2.4221	0.9888	36.1248	41.2085	34.00	40.00
AH Plus, 24 hours	10	4.500	0.4472	0.1825	4.0307	4.9693	4.00	5.00
AH Plus, 48 hours	10	0	0	0	0	0	0	0
AH Plus, 7 days	10	0	0	0	0	0	0	0
GuttaFlow2, 24 hours	10	0	0	0	0	0	0	0
GuttaFlow2, 48 hours	10	0	0	0	0	0	0	0
GuttaFlow2, 7 days	10	0.000	0	0	0	0	0	0

**Figure 4:**
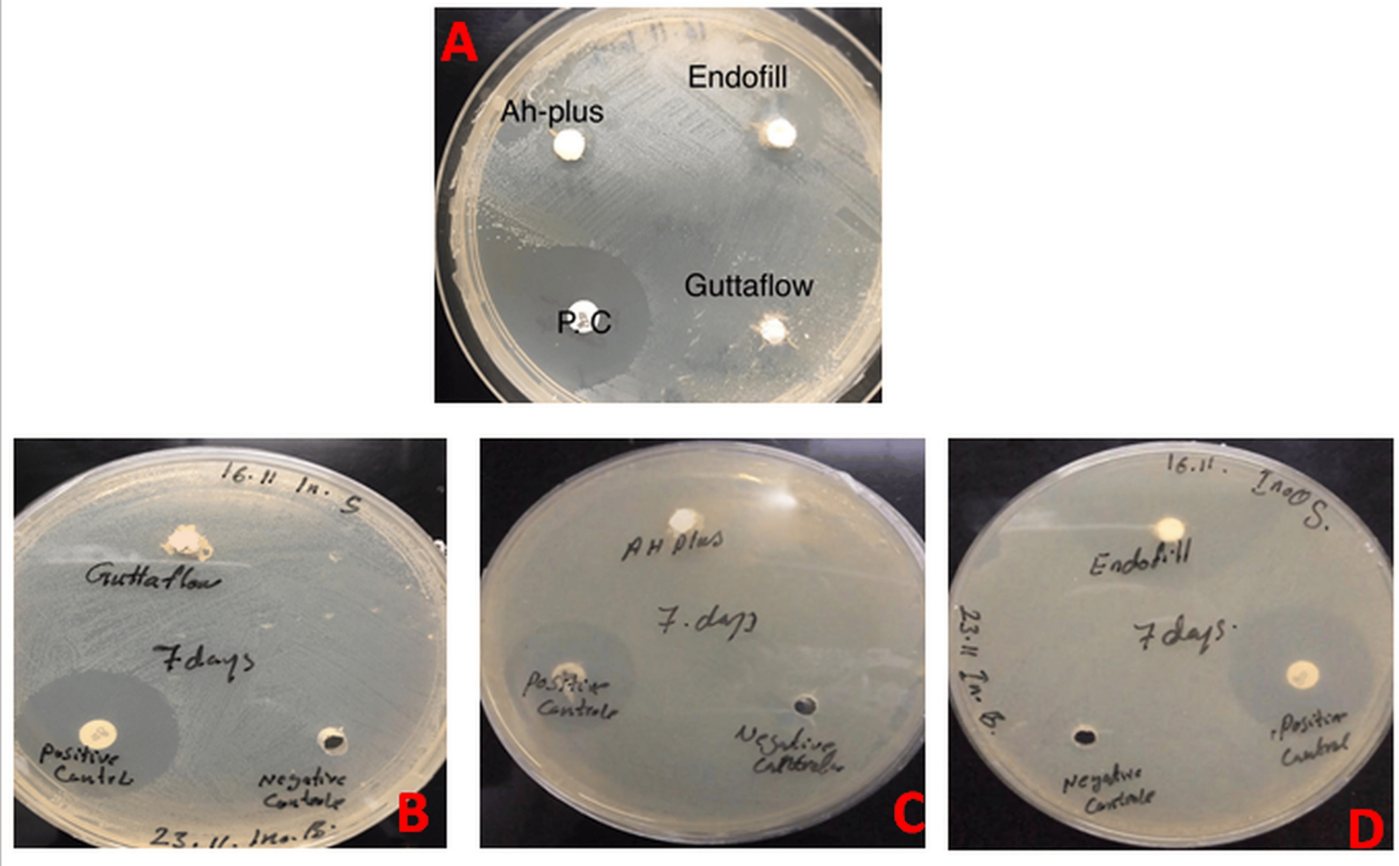
Antibacterial test (inhibition zones). (A) Inhibition zones after 24 hours. (B-D) Inhibition zones after seven days.

In the first 24 hours, the Endofill and AH Plus sealer groups showed antibacterial activity, whereas the GuttaFlow2 sealer group exhibited no inhibition zones. There was a statistically significant difference between the tested groups (p < 0.05), as shown in Table 2. After 48 hours of mixing, only the Endofill sealer demonstrated antibacterial activity, with no inhibition zones observed for AH Plus and GuttaFlow2 (p < 0.05). The same result was observed after seven days of mixing, where only the Endofill sealer showed an antibacterial effect, with a diameter of inhibition zones measuring 38.6667 mm, compared to AH Plus and GuttaFlow2, which showed no inhibition zones (p < 0.05). The diameter of the inhibition zone for the Endofill sealer group increased significantly over time, from 24 hours to seven days after mixing, as shown in Figure 4.

**Table 2:** Post hoc multiple comparisons of agar diffusion test of the diameter of inhibition zones of tested groups at different time intervals (24 hours, 48 hours, and seventh day of mixing). * The mean difference is significant at the p < 0.05 level.

Time intervals, sealer type	At 24 hours		
	Endofill	AH Plus	GuttaFlow2
Endofill	-	0.000*	0.000*
AH Plus	0.000*	-	0.000*
GuttaFlow2	0.000*	0.000*	-
	At 48 hours		
Endofill	-	0.000*	0.000*
AH Plus	0.000*	-	1.000
GuttaFlow2	0.000*	1.000	-
	At the 7th day of mixing		
Endofill	-	0.000*	0.000*
AH Plus	0.000*	-	1.000
GuttaFlow2	0.000*	1.000	-

Flow test

In this study, the maximum flow value was observed in the GuttaFlow2 group (34.020 mm), and the lowest was in the Endofill group (29.080 mm). The flow value for AH Plus was 33.920 mm (Table 3). Significant differences were observed in flow values between the Endofill group and both the GuttaFlow2 and AH Plus groups (p < 0.05) (Table 4). However, there was no statistically significant difference in flow values between the GuttaFlow2 and AH Plus groups (p = 0.609).

**Table 3:** ANOVA test of flow test.

Samples	N	Mean	SD	S. error	95% confidence interval for mean		Min	Max
					Lower bound	Upper bound		
Endofill	10	29.080	0.5287	0.1672	28.7018	29.4582	28.20	29.70
AH Plus	10	33.920	0.2699	0.0853	33.7269	34.1131	33.60	34.30
GuttaFlow2	10	34.020	0.4565	0.1443	33.6934	34.3466	33.00	34.60

**Table 4:** Post hoc multiple comparisons of flow test (LSD test) * The mean difference is significant at the p < 0.05 level. LSD: least significant difference.

(I) group	(J) group	Mean difference (I-J)	S. error	Sig.	95% confidence interval	
					Lower bound	Upper bound
Endofill	AH Plus	-4.8400*	0.1933	0.000	-5.2368	-4.4432
	GuttaFlow2	-4.9400*	0.1933	0.000	-5.3368	-4.5432
AH Plus	Endofill	4.8400*	0.1933	0.000	4.4432	5.2368
	GuttaFlow2	-0.1000	0.1933	0.609	-0.4968	0.2968
GuttaFlow2	Endofill	4.94000*	0.1933	0.000	4.5432	5.3368
	AH Plus	0.10000	0.1933	0.609	-0.2968	0.4968

## Discussion

The purpose of endodontic sealers in root canal treatment is to fill the space between the obturating material and the root canal dentinal wall, reducing leakage to lower the risk of infection by remaining microbes. Favorable antimicrobial activity is highly desirable for an ideal endodontic root canal sealer. *E. faecalis* is a common microorganism observed in persistent asymptomatic endodontic infections, with its prevalence in these infections ranging from 24% to 77% [11].

This study compared the antimicrobial activities of three endodontic sealers: Endofill, AH Plus, and GuttaFlow2. The null hypothesis is rejected, as there are significant differences between the tested sealers at different time periods (p < 0.05).

In the present study, the Endofill sealer showed the highest antibacterial activity compared to all other groups. The results demonstrated that all tested samples in the Endofill sealer group exhibited antimicrobial activities at different time intervals, with activity increasing significantly from 24 hours to 48 hours, and the largest inhibition zones were recorded seven days after mixing the Endofill sealer. These findings are consistent with previous studies [12,13]. The studies by Hachem et al. and Rosa et al. concluded that ZOE sealers can significantly reduce the number of viable bacterial cells of *E. faecalis* [13,14].

The strong antibacterial activity of Endofill may be attributed to the release of free eugenol from the ZOE sealer, which is a phenolic compound effective against a wide range of microbial cells [15].

Despite outstanding dimensional stability and low solubility for a durable, safe seal of AH Plus, the existing fact regarding the inability of bonding to gutta percha to form a hybrid layer is considered a major shortcoming of this material, hence the results from this study also show that the AH Plus group exhibited bactericidal activity only during the first 24 hours after mixing. This finding aligns with the studies by Sagsen et al. and Zhang et al., who also reported that freshly mixed AH Plus had greater antimicrobial effects at the first five to 20 minutes [8,16]. The possible antibacterial effects of AH Plus could be due to the release of formaldehyde during the setting process of resin-based sealers [17]. Other studies have indicated that the antimicrobial activity of resin-based sealers may also be related to bisphenol A diglycidyl ether [18]. The unpolymerized components (epoxide and amine) may be released into the surrounding environment during the polymerization process, which could explain the initial strong antimicrobial effects [19].

The results of the present study were consistent with those of Zhang et al. [16] and Kayaoğlu et al. [20], who found that freshly mixed AH Plus effectively killed *E. faecalis*. However, AH Plus samples tested at 48 hours and seven days after mixing showed no antimicrobial activity against *E. faecalis*, which is in agreement with a previous study by Bailón-Sánchez et al. [21], who found AH Plus to be ineffective against *E. faecalis*. Additionally, Kangarlou et al. [22] showed that freshly mixed AH Plus had powerful antimicrobial effects against *E. faecalis*; however, no antimicrobial effects were found after 24 hours, 48 hours, and seven days. Zhang et al. also demonstrated that the AH Plus sealer lost nearly all of its antimicrobial activity after 24 hours [16]. This critical drop in the antimicrobial activity of AH Plus after 48 hours and seven days of setting is attributed to the fully set sealer losing its capability to release antimicrobial agents [22].

The results of the present study show that GuttaFlow2 did not exhibit any antimicrobial activity in the agar diffusion test at 24 hours, 48 hours, and seven days of setting. GuttaFlow2 contains micro-silver particles as the antimicrobial ingredient, as an alternative to nano-silver. Micro-silver has a larger size and smaller surface area compared to nano-silver, resulting in fewer atoms available for biochemical interactions with microbes, which leads to reduced antimicrobial activity [23]. Various factors, such as shape, particle size, water chemistry, and stability, can affect the antibacterial activity of silver nanoparticles [24]. The results of the present study are consistent with previous studies that revealed minimal antibacterial effects of GuttaFlow2 [9,24,25].

Appropriate flowability of endodontic sealers is a favorable feature, as it helps fill the gap between the root canal wall and gutta-percha and allows access to lateral canals [26]. Additionally, adequate flow enhances the distribution of sealers into the complex anatomy of the root canal, which is challenging to access and disinfect [27].

All the sealer groups exhibited flow values greater than the minimum recommended by the American National Standards Institute/American Dental Association (ANSI/ADA) (20 mm). The highest flow values were observed in the GuttaFlow2 sealer group, followed by the AH Plus group, while the Endofill group demonstrated the lowest flow values. The high flow values of GuttaFlow2 can be attributed to the reaction between the polydimethylsiloxane, paraffin, and silicone oil it contains, as well as the pressure applied by the glass plate during the flow test. These sealers have thixotropic properties under compression, which enhance their fluidity and penetration [28,29].

The flow values of the AH Plus group are due to its composition, as epoxy resin is the key ingredient responsible for the flow of resin-based endodontic sealers. Mert et al. studied the flow of five root canal sealers and demonstrated that the determining factor for flow may not be the formulation, but rather the end density and setting reaction [30].

Limitations of the study

In vitro studies have limitations as these types of studies may not fully represent clinical scenarios; additionally, in vitro studies do not accurately reflect the real world, indicating potential discrepancies between laboratory findings and actual clinical conditions. Moreover, in vitro studies often exhibit suboptimal methodological quality, with low confidence in the results due to weaknesses in critical and non-critical assessment items. These drawbacks underscore the importance of complementing and supporting the in vitro studies with robust in vivo clinical trials to ensure and increase the value of the efficacy and reliability of the obtained results. Accordingly, clinical trials and long-term follow-up studies using various types of sealers over extended periods would be highly valuable for evaluating the clinical performance of these sealers.

## Conclusions

Endofill achieved the highest antibacterial activity at all three tested time intervals. In contrast, GuttaFlow2 showed no antimicrobial activity, while AH Plus demonstrated antimicrobial effects only during the first 24 hours of mixing and lost its antibacterial activity after setting. All evaluated root canal sealers exhibited adequate flow capacities that meet the requirements of the International Organization for Standardization (ISO) 6876:2012 specifications. Despite its highest antibacterial activity, Endofill had the lowest flow values compared to the other groups, whereas GuttaFlow2 and AH Plus demonstrated the highest flow values.
